# Identification of a novel missense mutation of *MIP* in a Chinese family with congenital cataracts by target region capture sequencing

**DOI:** 10.1038/srep40129

**Published:** 2017-01-06

**Authors:** Bo Jiang, Yanhua chen, Baisheng Xu, Nan Hong, Rongrong Liu, Ming Qi, Liping Shen

**Affiliations:** 1 Department of Ophthalmology, the First Affiliated Hospital, Zhejiang University School of Medicine, Hangzhou, China; 2 BGI-Shenzhen, Shenzhen, China; 3 School of Bioscience and Bioengineering, South China University of Technology, Guangzhou, China; 4 Casey Eye Institute Molecular Diagnostic Laboratory, Portland, Oregon, USA; 5 Department of Ophthalmology, Tongde Hospital of Zhejiang Province, Hangzhou, China; 6 Division of Hematology-oncology, Children’s Hospital of Zhejiang University School of Medicine, Hangzhou, China; 7 Department of Cell Biology and Medical Genetics, Zhejiang University School of Medicine, Hangzhou, China; 8 Department of Pathology and Laboratory of Medicine, University of Rochester Medical Centre, Rochester, New York, USA

## Abstract

Congenital cataract is both clinically diverse and genetically heterogeneous. To investigate the underlying genetic defect in three-generations of a Chinese family with autosomal dominant congenital cataracts, we recruited family members who underwent comprehensive ophthalmic examinations. A heterozygous missense mutation c.634G > C (p.G212R) substitution was identified in the *MIP* gene through target region capture sequencing. The prediction results of PolyPhen-2 and SIFT indicated that this mutation was likely to damage the structure and function of *MIP*. Confocal microscopy images showed that the intensity of the green fluorescent signal revealed much weaker signal from the mutant compared to the wild-type MIP. The expressed G212R-*MIP* was diminished and almost exclusively cytoplasmic in the HeLa cells; whereas the WT-*MIP* was stable dispersed throughout the cytoplasm, and it appeared to be in the membrane structure. Western blot analysis indicated that the protein expression level of the mutant form of *MIP* was remarkably reduced compared with that of the wild type, however, the mRNA levels of the wild-type and mutant cells were comparable. In conclusion, our study presented genetic and functional evidence for a novel *MIP* mutation of G212R, which leads to congenital progressive cortical punctate with or without Y suture.

Congenital cataracts are the leading cause of childhood blindness. They occur in 1–6 cases per 10,000 live births in industrialized countries, and of 5 to 15 per 10,000 in the poorest areas of the world^1^,^2^,^3^,^4^. About 20,000–40,000 new cases of bilateral congenital cataracts are diagnosed each year^4^. Between 8.3 and 25% of congenital cataracts are believed to be inherited, and the lens alone may be involved, accounting for approximately 70% of congenital cataracts^5^,^6^.

Congenital cataracts are most frequently inherited as autosomal dominant traits, although autosomal recessive, and X-linked inheritance exist^7^. To date, more than 20 genetic loci have been linked to autosomal dominant inherited cataracts. Among these, the identified genes encode crystallines, membrane transport and channel proteins, cytoskeletal proteins, and some growth and transcription factors^6^,^8^.

Major intrinsic protein (*MIP*), also known as *Aquaporin 0* (*AQP0*), is an intrinsic membrane protein, that comprises approximately 50% of the total integral fiber cell membrane proteins in the lens^9^. *MIP* is initially expressed as the first primary fibers begin and as the secondary fibers differentiate, then continually undergoes posttranslational modifications during differentiation and aging^10^. It plays a critical role in lens-specific water channels. To date, several mutations in *MIP* have been linked to mouse and human congenital cataracts. In the present study, we identified the molecular and functional defects in three generations of a Chinese family with autosomal dominant congenital cataracts (ADCC), and detected a novel c.634G > C transition in exon 4 of the *MIP* gene by target region capture sequencing.

## Results

### Clinical features

**Figure 1:**
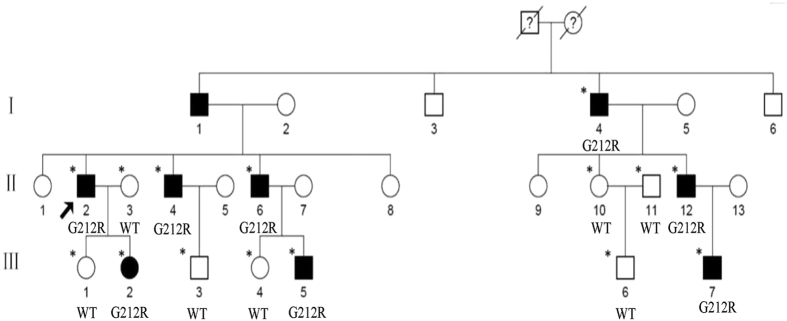
Pedigree of the family. Squares and circles indicate males and females, respectively. Black symbols indicate affected members and open symbols indicate unaffected individuals. The diagonal line indicates a deceased family member and the black arrow indicates the proband. Asterisks indicate sequenced samples. A missense mutation c.634G > C (p.G212R) was identified in affected members (G212R). WT indicated wild type.

**Figure 2:**
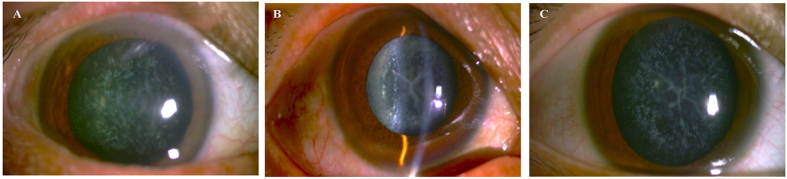
Slit lamp photographs of the patients. (**A**) the proband (II:2) showed a punctate cataract; (**B**) his younger brother (II:6) and (**C**) the brother’s son (III:5) showed Y-suture cataracts combined with punctate cortical opacities.

We identified a Chinese family with three generations of individuals (eight affected individuals and seven unaffected individuals) with diagnoses of ADCC (Fig. 1). The proband (II:2) was a 60-year-old male with a complaint of blurred vision in both eyes who underwent bilateral cataract surgery in our hospital in January and April 2013. The other affected participants of the family were aged 20–77 years, and manifested bilateral fine punctate anterior and posterior cortical opacities, combined with Y-sutural cataracts. However, the proband had a different type of cataract with fine punctate cortical opacities. None of the younger affected patients (III:2, III:5, III:7) complained of significant visual deterioration, and they were unaware of their cataracts until the examinations. The punctate opacities increased in number and both the punctate and Y-sutural opacities became gradually denser with age (Fig. 2). There were no other ocular or systemic abnormalities or symptoms.

### Capture panel sequencing results, variant analysis and validation

**Figure 3:**
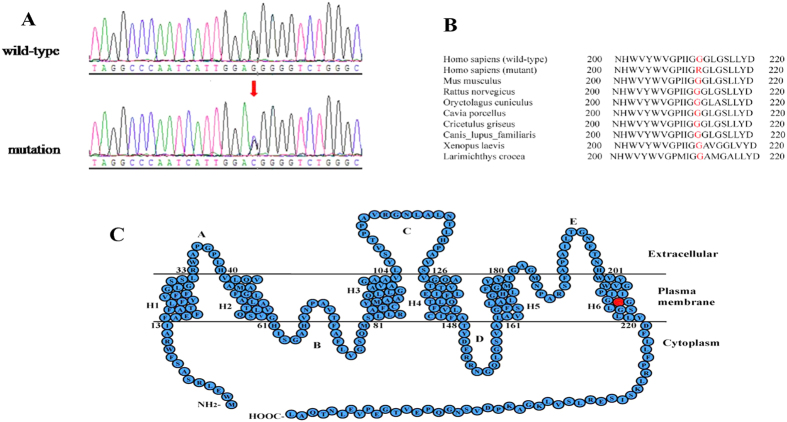
Mutation analysis of the *MIP* gene. (**A**) Sequencing results of *MIP* from unaffected and affected members of the family. The position of nucleotide substitution is indicated by a red arrow. (**B**) Phylogenetic conservation analysis. The result of a multiple-sequence alignment from various species showed that the Glycine at position 212 of *MIP* is highly conserved (marked in red). (**C**) A schematic diagram of the topology of *MIP*. Amino acid residue 212 is located within the 6th transmembrane domain (red circle).

Using the capture panel described in the Methods, an average of 137× and 188× depth in the target region was achieved, and 96.49% and 97.08% of designed target regions were covered by at least 20× in the proband and his brother, respectively. This demonstrated that sufficient data quality was achieved to identify variants. In total, 723 and 744 variants were detected in the coding regions and adjacent intronic regions in the proband and his affected brother, 57 and 52 of which were rare (the variants were filtered out if their frequency was >0.01 in 1000 the genome database, dbSNP, HapMap project or local database). Only four rare variants were found in the 42 known congenital cataract causing genes in both samples (Table 1). Two of the four rare variants were in the *LEPREL1* gene, which has been reported to be an autosomal recessive gene mainly cause myopia^11^,^12^. It seems not to have affected the patients as the disease in the inherited in a autosomal in this family exhibits an dominant pattern and does not show myopia. The heterozygous variant c.10–7C > G in the *CRYGD* gene is in a potential accept splice site, but was predicted as a polymorphism by Mutation Taster (http://www.mutationtaster.org/). Direct sequencing of the variant of CRYGD gene was done in samples of the family members, and the results indicated the heterozygous variant c.10–7C > G in the CRYGD gene only exhibited in the proband (II:2) and his affected brother (II:4). There was no cosegregation of the variant of CRYGD gene with affected members in this family. Therefore, it seems not to be a disease-causing mutation. The c.634G > C mutation of the *MIP* gene, which has been reported to cause autosomal dominant cataracts^13^,^14^, was a novel heterozygous missense mutation. This mutation changed Glycine (GGG) to Arginine (CGG) at position 212 (p.G212R) (Fig. 3A). Segregation analysis was performed, and this mutation cosegregated well with all affected participants and was not found in unaffected members or the 100 unrelated normal controls.

### Bioinformatics analysis

The mutation was predicted to be probably damaging by PolyPhen-2 with a score of 0.958 (sensitivity: 0.63; specificity: 0.92), and to affect protein function by SIFT, with a score of 0.00 (media information conservation 3.01). Collectively, these results strongly indicated that the p.G212R mutation is likely to be deleterious to the protein and may therefore be responsible for the congenital cataracts. The result of a multiple sequence alignment showed that the Glycine at position 212 of *MIP* is highly conserved among various species (Fig. 3B).

### Result of Fluorescence Microscopy analysis

**Figure 4:**
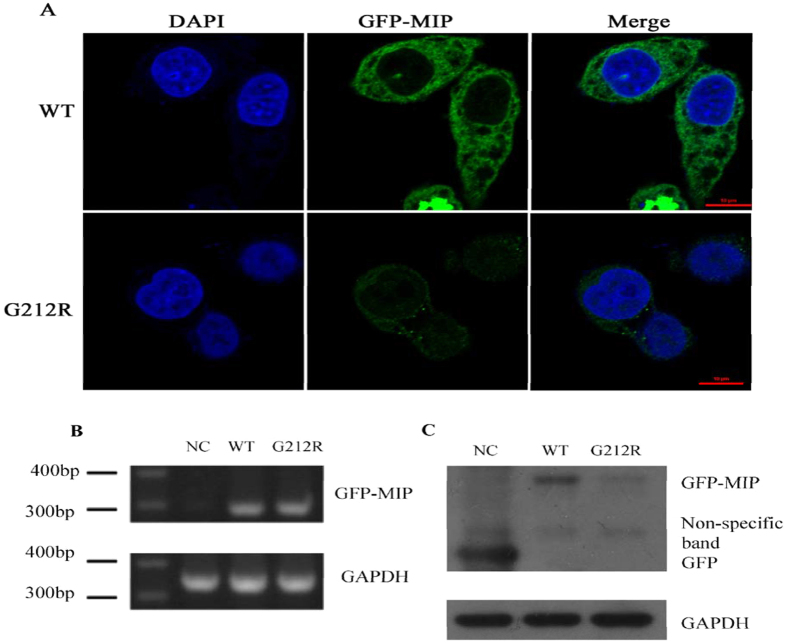
mRNA and protein levels of WT-*MIP* and G212R-*MIP*. (**A**) Expression of WT and G212R -*MIP* proteins in transfected HeLa cells viewed by confocal microscopy. The green fluorescence was much weaker in HeLa cells transfected with the G212R construct than in those transfected with the wild type construct. The expressed G212R-*MIP* was diminished and almost exclusively cytoplasmic in the cells. In contrast, WT-*MIP* was stable dispersed throughout the cytoplasm, and it appeared to be in the plasma membrane, in the subcellular organelles and in the nuclear membrane. (**B**) The RT-PCR revealed no difference in the *MIP* mRNA level between WT-*MIP* and G212R-*MIP*. (**C**) Western blot analysis indicated that the G212R mutation significantly reduced the protein levels of *MIP*, consistent with the expression level of the green fluorescence. GAPDH was used as the control.

We investigated the expression of WT-MIP and G212R-MIP after the transient transfection of HeLa cells. The green fluorescence was much weaker in HeLa cells transfected with the G212R construct than in those transfected with the wild type construct. The expressed G212R-MIP was diminished and almost exclusively cytoplasmic in the cells. In contrast, WT-MIP was stable dispersed throughout the cytoplasm, and it appeared to be in the plasma membrane, in the subcellular organelles and in the nuclear membrane (Fig. 4A).

### Results of RT-PCR and Western blot analysis

RT- PCR was used to measure the RNA transcription level of WT-*MIP* and G212R-*MIP* after total RNA was extracted from the HeLa cells. The RT-PCR revealed a similar relative *MIP* mRNA expression levels in WT-*MIP* and G212R-*MIP* (Fig. 4B). However, Western blot analysis indicated that the G212R mutation significantly reduced protein expression levels of *MIP*, consistent with the green fluorescence signal intensity (Fig. 4C), indicating that the mutation decreased the protein production of *MIP* gene.

## Discussion

In the present study, we investigated the genetic and functional defects of three generations of a Chinese family mainly affected with a typical Y-suture cataract combined with punctate cortical opacities. Target region capture sequencing revealed a novel missense mutation of the *MIP* gene in exon 4 at nucleotide 634, which caused a Glycine-to-Arginine substitution at 212 position (p.G212R).

To date, 16 mutations in the *MIP* gene have been reported to be associate with autosomal dominant cataracts, including 10 missense mutations^8^,^13^,^15^,^16^,^17^,^18^,^19^,^20^,^21^; one acceptor splice-site mutation^22^; one donor splice-site mutation^23^; one deletion that causes a frameshift at 638delG^14^; one initiation codon mutation^24^; and two nonsense mutations^25^,^26^. The cataract phenotypes are significantly different among the *MIP* mutation families. Phenotypically identical cataracts can result from mutations at different genetic loci and may have different inheritance patterns, while phenotypically variable cataracts can be found in a single large family^6^. In the family, the proband had a different type of cataract as compared with other affected family members although they had the same mutation. We compared the phenotype of our family with other reported families and found that the clinical features were very similar to those reported in other studies^14^,^25^: all manifested as fine punctate opacities in the cortex and Y suture. The cataract family reported by Yu *et al*.^25^ was associated with a nonsense mutation (c.337C > T (p.R113X)) that produced a severely truncated protein. Geyer *et al*.^14^ reported a single nucleotide deletion, that caused a frameshift and a premature stop codon that truncated six amino acids from the C-terminus of *MIP*. This family had different cataract phenotypes including both fine punctate opacities in the cortex and Y sutures, and fine white punctate opacities in the cortex, similar to our cataract family.

The *MIP* gene encodes a 28-kDa protein with 263 amino acids, and it is primarily and abundantly expressed in the lens. After Peter Agre’s laboratory developed a functional assay for water channels, the *MIP* family became the aquaporin family and *MIP* became known as aquaporin 0^10^. Besides functioning as a water channel, *MIP* also plays a role in interfiber adhesion and intracellular interaction with lens fiber proteins, being required for lens transparency and accommodation. Mutations in the *MIP* gene in human and mice results in genetic cataracts; deletion of the *MIP*/*AQP0* gene in mice results in dominant cataracts, decrease of water channel activity and a lack of suture formation required for maintenance of the lens fiber architecture^10^,^27^. As an intrinsic membrane protein, MIP inserts in the plasma membrane with six transmembrane bilayer-spanning domains (H1–H6), resulting in three extracellular loops (A, C and E), two intracellular loops (B and D), and the N- and C-terminal intracellular domains. It assembles as a tetramer with four water pores in the endoplasmic reticulum (ER) before being transported and inserted into the plasma membrane. Each monomer is a water channel and can function independently^28^,^29^. The first missense mutation in H6 of *MIP* (c.644G > A) was reported to be associated with punctate cataracst in 2014^8^; this is the second substituation mutation in H6 that can cause ADCC.

In the present study, our data suggest that the c.634G > C, G212R substitution may contribute to ADCC pathogenesis in this family. First, we investigated the expression of WT and G212R -MIP proteins in transfected HeLa cells viewed by confocal microscopy. The remarkable difference in the green fluorescence signal intensity indicated that the production of the mutant protein decreased. The expressed WT-*MIP* was stable dispersed throughout the cytoplasm, and it appeared to be in membrane structure. In contrast, G212R-*MIP* was diminished and almost exclusively cytoplasmic in the cells. Second, immunoblot analysis indicated that the protein expression level of the mutant form of *MIP* was remarkably reduced compared with that of the wild type, however, the mRNA levels of the wild-type and mutant cells were comparable. These results were similar to previous p.G165D results^20^. All the above results indicated the mutation resulted in instability of the protein. The loss of the water channel function could reflect RNA instability, impaired translation, protein degradation, or differential post-translational modification as well as impaired trafficking. Several mutations have been functionally characterized *in vitro*. The p.E134G^13^, p.T138R^13^, p.G165D^20^, and p.G215D^8^ mutations may result in the loss of water permeability due to impaired trafficking of mutant proteins to the plasma membrane. The 638G deletion resulted in the impairment of the cell membrane, localizing the mutant protein in the endoplasmic reticulum without trafficking it to the plasma membrane, and inducing cellular cytotoxicity^30^. Retention or accumulation of proteins in the ER can cause ER stress and an unfolded protein response, which have been implicated in the pathogenesis of several diseases^20^.

Target region capture sequencing is a combination of genomic region enrichment and next-generation sequencing technology. The compatible performance in capture coverage, as well as the lower cost and shorter time required, makes it a good choice for clinical diagnosis of a heterogeneous group of monogenic disorders^31^.

In summary, through target region capture sequencing, this study reported ADCC caused by a novel missense mutation c.634G > C in exon 4 of the *MIP* gene, resulting in a Glycine -to-Arginine (p.G212R) substitution in the H6 domain of *MIP*. Moreover, this study presented evidence that p.G212D caused the production of the mutant protein to be reduced and retained in the cytoplasm. Further studies are needed to elucidate the pathophysiologic changes caused by this mutation. This study expands the spectrum of mutations that cause congenital cataracts.

## Methods

### Clinical data and genomic DNA preparation

Three-generations of a Han Chinese family from Zhejiang Province with ADCC were recruited through the Department of ophthalmology, the first Affiliated Hospital, Zhejiang University School of Medicine, Hangzhou, China. All study participants underwent detailed clinical and ophthalmological examinations. This study was approved by the Institutional Review Board of the First Affiliated Hospital, Zhejiang University School of Medicine, and adhered to the Tenets of the Declaration of Helsinki. Each participant was informed about the nature of the study, and written informed consent was obtained. Genomic DNA was extracted from the peripheral blood for PCR amplification using the QIAamp DNA Blood Mini Kit (Qiagen, Hilden, Germany) according to the manufacturer’s standard procedure. A total of 100 ethnically matched subjects without a family history of congenital cataracts were recruited as controls.

## Target region capture sequencing and analysis

### Library preparation and target region capture sequencing

A custom-made capture panel (BGI, Shenzhen China) was designed to capture 351 genetic eye disease genes (gene list not shown), which included 42 inheritable genetic congenital cataract-related genes (Supplemental Table 1). These genes were collected from OMIM (http://www.ncbi.nlm.nih.gov/omim/) and the published literatures. The genomic DNA of the proband (II:2) and his affected brother (II:4) was separated into approximately 200–300 base pair (bp) fragments and used to generate a paired-end library (Covaris S2, Woburn, MA, USA). The library capture was completed through BGI using the custom-made capture array and sequenced on an Illumina HiSeq2000 Analyzer (San Diego, CA). Image analysis and base calling were performed using the Illumina Pipeline to generate raw data.

### Variant identification and validation

To detect the variants in the patients, we applied filtering criteria to generate clean reads, and then aligned the clean reads to the human genome reference from the NCBI database (NCBI build 37.1) using the Burrows Wheeler Aligner (BWA) Multi-Vision software package. Single-nucleotide variants (SNVs) and insertions and deletions (InDels) were extracted by the Genome Analysis Tool Kit (GATK). All SNVs and InDels were annotated using the NCBI dbSNP, HapMap project, 1000 Genome Project and the BGI local database of a 1000 healthy adults. The candidate variations of known congenital cataract genes were validated by polymerase chain reaction (PCR) and Sanger sequencing. PCR primer sets were designed via Primer Premier 6.0 (*MIP* forward 5′-GTG ACC TCT GGT TGT CCA TTG G-3′; *MIP* reverse 5′-GCT AAG GTG TGG GAT AAA GGA GTA A-3′; CRYGD forward 5′-GCC AGT GAT AGC AAT CCG AAT-3′; CRYGD reverse 5′-AAC CAT CCA GTG AGT GTC CT-3′), and products were sequenced using a Bigdye terminator v3.1 cycle sequencing kit (ABI, Foster City, CA, USA) and analyzed on an ABI 3700XL Genetic Analyzer.

### Bioinformatics analysis

The online PolyPhen-2 and SIFT programs were used to predict whether this amino acid substitution affected the structure and function of the protein. The amino acid sequences of MIP from several different species were obtained from NCBI GenBank, and conservation analysis was performed using MEGA 7.

### Plasmid construction

The wild type *MIP* (WT-*MIP*) gene was synthesized by GenScript (Nanjing, China), and pEGFP-WT resultant plasmids were constructed to create EGFP-*MIP* fusion proteins. The expression vector for G212R mutant *MIP* (G212R-*MIP*) was constructed using site-directed mutagenesis with the following primers: forward primer 5′-GGC CCA ATC ATT GGA CGG GGT CTG GG-3′; reverse primer 5′-GTC CAA TGA TTG GGC CTA CCC AGT AC-3′. The mutation was confirmed through DNA sequencing.

### Cell culture and transfection

HeLa cells were cultured in Dulbecco’s modification of Eagle’s medium (DMEM) supplemented with 10% fetal bovine serum (FBS) in a 37 °C incubator with 5% CO_2_. Transfection was performed Lipofectamine 2000 (Invitrogen Corporation, Carlsbad, CA, USA) according to the manufacturer’s protocol.

### Fluorescence Microscopy analysis

HeLa cells were plated in 6-well plates 24 h prior to transfection at approximately 60% confluency. Twenty four hours after transfection, the cells were washed with PBS, fixed with 4% paraformaldehyde in PBS for 15 min, and permeabilized with 0.2% Triton X-100 for 20 min; the nuclei were stained using 1 mg/mL DAPI for 15 min. The cells were analyzed using a NIKON A1R confocal microscope (Japan).

### Reverse transcription - polymerase chain reaction (RT-PCR) analysis

Total RNA was extracted from the HeLa cells 24 h after transfection using TRIZOL^TM^ (Invitrogen) reagent, in accordance with standard procedures. RNA was reverse-transcribed using a PrimeScript RT reagent Kit with gDNA Eraser (Perfect Real Time, TaKaRa, Otsu, Shiga, Japan) according to the manufacturer’s instructions. The primer sequences for RT-PCR were: forward primer 5′-CTA TGG CAT TTG GCT TGG-3′; reverse primer 5′-GAA CTG GAG CGT CAG GAA G-3′. GAPDH gene as an internal control was assessed with forward primer 5′-CCA TCA CCA TCT TCC AGG AG-3′; and reverse primer 5′-GGC CAT CCA CAG TCT TCTG G-3′. PCR products were separated by electrophoresis on 2% agarose gels and confirmed by sequencing.

### Western blot analysis

After transfection with WT-*MIP* or G212R-*MIP* plasmids separately, HeLa cells were harvested and lysed in RIPA lysis buffer. Total proteins were extracted and separated by 10% SDS/PAGE gels, and transferred to PVDF membranes separately. The membranes were incubated with antibodies of anti-GAPDH and anti-GFP (Proteintech Group, Wuhan). The signals were visualized using chemiluminescent detection.

## Additional Information

**How to cite this article**: Jiang, B. *et al*. Identification of a novel missense mutation of *MIP* in a Chinese family with congenital cataracts by target region capture sequencing. *Sci. Rep.* **7**, 40129; doi: 10.1038/srep40129 (2017).

**Publisher's note:** Springer Nature remains neutral with regard to jurisdictional claims in published maps and institutional affiliations.

## Supplementary Material

Supplementary Table 1

## Figures and Tables

**Table 1:** Rare variants in congenital cataract causing genes.

Location	Gene	NM_ID	DNA Change	Residue Change	Het/Hom	Function	Freq_1kgenome*	Freq_local**
Proband (II:2)								
2q33.3	CRYGD	NM_006891.3	c.10–7C > G	—	Het	intron	0.003	0.002224
3q28	LEPREL1	NM_001134418.1	c.1439A > G	p.Lys480Arg	Het	missense	0	0
3q28	LEPREL1	NM_001134418.1	c.1006–4_1006-3insT	—	Het	intron	0	0
12q13.3	MIP	NM_012064.3	c.634G > C	p.Gly212Arg	Het	missense	0	0
13q12.11	GJA3	NM_021954.3	c.531G > C	—	Het	synonymous	0	0
His brother (II:4)								
2q33.3	CRYGD	NM_006891.3	c.10–7C > G	—	Het	intron	0.003	0.002224
3q28	LEPREL1	NM_001134418.1	c.1439A > G	p.Lys480Arg	Het	missense	0	0
3q28	LEPREL1	NM_001134418.1	c.1006–4_1006-3insT	—	Het	intron	0	0
12q13.3	MIP	NM_012064.3	c.634G > C	p.Gly212Arg	Het	missense	0	0

^*^Freg_1kgenome means frequency of this variation in 1 k genome project.

^**^Freq_local means the frequency of this variation in BGI’s local database (100 normal people.
