# Supplementary material for: Identification of a novel missense mutation of MIP in a Chinese family with congenital cataracts by target region capture sequencing
Source: Sci Rep. 2017 Jan 6;7:40129. doi: 10.1038/srep40129 (PMC5216388; doi:10.1038/srep40129)
Supplement: Supplementary Table 1 [file srep40129-s1.rtf]

Identification of a novel missense mutation of MIP in a Chinese family with congenital cataracts by target region capture sequencing
Bo Jiang 1,a, Yanhua chen 2,3,4,a, Baisheng Xu 5, Nan Hong 1, Rongrong Liu 6, Ming Qi 7, 8,*, Liping Shen1,**
1 Department of Ophthalmology, the First Affiliated Hospital, Zhejiang University School of Medicine, Hangzhou, China
2 BGI-Shenzhen, Shenzhen, China
3 School of Bioscience and Bioengineering, South China University of Technology, Guangzhou, China
4 Casey Eye Institute Molecular Diagnostic Laboratory, Portland, Oregon, USA
5 Department of Ophthalmology, Tongde Hospital of Zhejiang Province, Hangzhou, China
6 Division of Hematology-oncology, Children's Hospital of  Zhejiang University School of Medicine, Hangzhou, China
7 Department of Cell Biology and Medical Genetics, Zhejiang University School of Medicine, Hangzhou, China
8 Department of Pathology and Laboratory of Medicine, University of Rochester Medical Centre, Rochester, New York, USA.

a The first two authors contributed equally to this work.
* Corresponding to: Ming Qi, Research Building A713, 866 Yuhangtang Road, Hangzhou, Zhejiang, 310058, China. Tel: +86 0571 88208274; 
** Corresponding to: Liping shen, 79 Qinchun Road, Hangzhou, Zhejiang, 310003, China. Tel.:+86 0571 87236791.
E-mail addresses:  mingqi@zju.edu.cn (Ming Qi ), shenlp@hotmail.com (Liping Shen)  


Suppementary table 1. List of 42 inheritable genetic congenital cataract-related genes.


2
